# 14-3-3ζ/TGFβR1 promotes tumor metastasis in lung squamous cell carcinoma

**DOI:** 10.18632/oncotarget.12690

**Published:** 2016-10-15

**Authors:** Yanbin Zhao, Wenbo Qiao, Xiaoyuan Wang, Hang Yin, Jianqi Cui, Yue Cui, Xuesong Chen, Jing Hu, Hailing Lu, Qingwei Meng, Yan Wang, Li Cai

**Affiliations:** ^1^ The Department of Internal Medical Oncology, Harbin Medical University Cancer Hospital, Harbin, Heilongjiang Province, China; ^2^ The Department of radiotherapy, Harbin Medical University Cancer Hospital, Harbin, Heilongjiang Province, China

**Keywords:** 14-3-3ζ, TGFβR1, metastasis, lung squamous cell carcinoma, EMT

## Abstract

14-3-3ζ is involved in tumor cell growth and apoptosis. However, the mechanism of 14-3-3ζ in lung squamous cell carcinoma (SCC) metastasis has not been illuminated. In our studies, we found that the expression of 14-3-3ζ was highly expressed in lung SCC compared to normal lung tissues. High expression of 14-3-3ζ was associated with pTNM stage (p<0.05) and lymph node metastasis (p<0.05). Furthermore, the expression of 14-3-3ζ protein was associated with high levels of TGFβR1 protein (p=0.005), and pSMAD3 (p=0.033). Lung SCC patients with high 14-3-3ζ expression have significantly shorter OS and DFS compared to patients with low 14-3-3ζ expression. Additionally, 14-3-3ζ knockdown inhibited cell proliferation, migratory and invasive properties of human lung SCC cells. TGFβR1 was involved in 14-3-3ζ-mediated cell proliferation and metastasis of lung SCC cells. Additionally, sh-14-3-3ζ can suppress tumor growth and metastasis *in vivo*. Thus, these data provide the evidence that 14-3-3ζ promote tumor metastasis and might be a prognostic biomarker and target for therapeutic strategy in lung SCC.

## INTRODUCTION

Lung cancer has the highest cancer morbidity and mortality in worldwide with a 5-year survival rate of 15% [1]. Histologically, lung squamous cell carcinoma (SCC) accounts for about 30-50% of all lung cancers. The survivaltime of lung SCC is still very short, though great efforts have been made in surgery, chemotherapy and radiotherapy [2]. The standard of adjuvant or first-line palliative chemotherapy remains platinum based doublet chemotherapy. To date, lung SCC is lack of effective targeted therapy compared with adenocarcinoma [3]. Thus, it is urgent that biomarkers are identified for monitoring of disease progression or prediction of prognosis of lung SCC. Furthermore, this may provide insightful information for the development of targeted molecular therapy and discovering the molecular mechanisms of lung SCC.

14-3-3ζ, a subtype of the 14-3-3 protein family, is observed to widely express in various cancers. It has been emphasized as a critical role in tumorigenesis and progression via interacting with cellular proteins such as intracellular signaling, cell transcription regulation, proliferation and apoptosis [4]. Dysregulation of 14-3-3ζ has been shown to contribute to tumorigenesis and cancer progression in several kinds of cancers, such as prostate cancer, breast cancer, liver cancer stem cells and oral cancer et al. [5–9]. With regard to lung cancer, it has been shown that 14-3-3ζ expression was up-regulated in NSCLC. Down-regulation of 14-3-3ζ in lung cancer cells increased sensitivity to cisplatin-induced cell death. Over expression of 14-3-3ζ and Hsp27 promoted NSCLC progression [10]. Those results implied that 14-3-3ζ might be involved in NSCLC chemosensitivity and progression.

As we known that the transforming growth factor β (TGFβ) superfamily is widely involved in cell growth, EMT and metastasis [11–12]. TGFβ receptor types 1 and 2 (TGFβR1 and TGFβR2), two types of cell-surface receptors, lead to the activation of TGFβ signaling to perform multiple intracellular functions. TGFβ binds its receptor TGFβR2. Then TGFβR2 recruits and causes phosphorylation of TGFβR1. TGFβR1 can phosphorylate the transcription factors Smad2 and Smad3, which can regulate the expression of down stream signaling genes, such as p21 and C-myc et al. In breast cancer cells, the binding of 14-3-3ζ protects TGFβR1 from degradation [13]. However, the correlation between 14-3-3ζ and TGFβR1 in lung SCC is not known. Interestingly, our results shown that the up-regulation of 14-3-3ζ were associated with increased TGFβR1 and Smad3 expression in lung SCC tissues.

In this study, we elucidated the mechanism of 14-3-3ζ mediated metastasis and prognosis in lung SCC. Compared with normal lung, the expression of 14-3-3ζ was highly expressed in lung SCC. Up-regulated expression of 14-3-3ζ was related to pTNM stage and the metastasis of lymph node. 14-3-3ζ regulated the EMT program and promoted cell metastasis through TGFβR1 in lung SCC. These findings revealed that 14-3-3ζ promote tumor metastasis and could be a prognostic biomarker and target for therapeutic strategy in lung SCC.

## RESULTS

### 14-3-3ζ is higher expressed in lung SCC tissues compared to normal tissues

**Figure 1:**
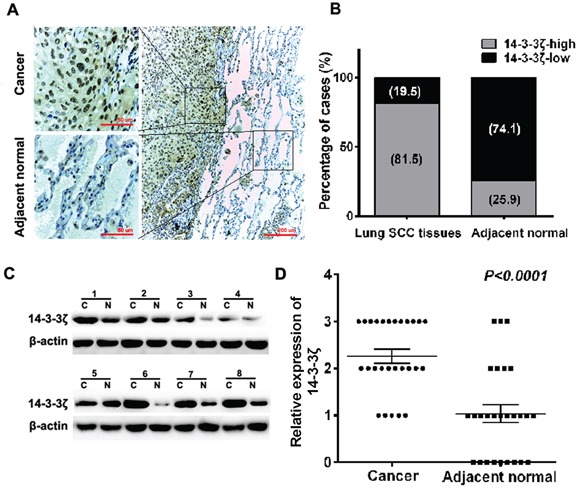
Expression of 14-3-3ζ is significantly up-regulated in lung SCC tissues compared with adjacent normal tissues by IHC and western blot **A.** IHC staining for 14-3-3ζ in human adjacent normal and lung SCC samples. Original magnification: 20× or 40×. High or low 14-3-3ζ expression was detected in cancerous or adjacent normal tissues, respectively. **B.** 14-3-3ζ expression patterns (high or low expression) were analyzed in human adjacent normal and lung SCC samples. **C.** Protein was extracted from matched lung SCC tissues and adjacent normal tissues and subjected to western blot analysis to examine 14-3-3ζ expression levels. β-Actin served as a loading control. **D.** The relative levels of 14-3-3ζ expression in lung SCC or adjacent normal tissue samples were quantified by western blot (1C) and normalized to β-actin (the internal control).

First, we investigated the expression of 14-3-3ζ in 27 pairs of human lung SCC samples and its adjacent normal tissues. 14-3-3ζ was mainly localized to the nuclei and the cytoplasm by IHC analysis. There are 81.5% (22/27) lung SCC tissues highly expressed 14-3-3ζ, compared with only 25.9% (7/27) adjacent normal tissues (*p*<0.0001) (Figures 1A and 1B). We further verified these results in 8 paired lung SCC samples using western blot analysis. Consistently, 14-3-3ζ was higher expressed in SCC samples (*p*<0.0001, Figure 1C and 1D). Our results proved that 14-3-3ζ is increased in lung SCC, which might promote tumorigenesis in lung SCC.

### The expression of 14-3-3ζ, TGFβR1 and pSMAD3 proteins in lung SCC tissues and the relationship with clinicopathological characteristics

We further assessed expression of 14-3-3ζ in 81 cases lung SCC tissues using IHC. The high expression of 14-3-3ζ was 55.6% in lung SCC samples (n=81). There was a significant association between 14-3-3ζ expression and advanced tumor TNM stages (*p*=0.031) and lymph node metastasis (*p*=0.004). While, 14-3-3ζ expression is not associated with other clinicopathological data (*p*>0.05) (Table 1).

**Table 1:** Association of three different proteins with clinicopathologic factors in 81 lung SCC tissues

Variables	No. of Patients	14-3-3ζ expression		*P* (χ^2^)	TGFβR1 expression		*P* (χ^2^)	pSMAD3 expression		*P* (χ^2^)
		positive (n,%)	negative (n,%)		positive (n,%)	negative (n,%)		positive (n,%)	negative (n,%)	
All cases	81	45(55.6)	36(44.4)		62(76.5)	19(23.5)		29(35.8)	52(64.2)	
Age (years)				0.282			0.441			0.386
≤55	18	8(44.4)	10(55.6)		15(83.3)	3(16.7)		8(44.4)	10(55.6)	
>55	63	37(58.7)	26(41.3)		47(74.6)	16(25.4)		21(33.3)	42(66.7)	
Gender				0.675			0.548			0.646
male	69	39(56.5)	30(43.5)		52(75.4)	17(24.6)		24(34.8)	45(65.2)	
female	12	6(50.0)	6(50.0)		10(83.3)	2(16.7)		5(41.7)	7(58.3)	
Tumor size(cm)				0.300			**0.002**			**0.023**
≤3	12	7(58.3)	5(41.7)		8(66.7)	4(33.3)		2(16.7)	10(83.3)	
3~5	48	18(37.5)	30(62.5)		42(87.5)	6(12.5)		23(47.9)	25(52.1)	
>5	21	11(52.4)	10(47.6)		10(47.6)	11(52.4)		4(19.0)	17(81.0)	
Differentiation				0.099			**0.006**			0.878
Well/moderate	26	11(42.3)	15(57.7)		15(57.7)	11(42.3)		9(34.6)	17(65.4)	
poor	55	34(61.8)	21(38.2)		47(85.5)	8(14.5)		20(36.4)	35(63.6)	
pT status				0.453			0.063			0.079
pT1	10	4(40.0)	6(60.0)		8(80.0)	2(20.0)		2(20.0)	8(80.0)	
pT2	59	33(55.9)	26(44.1)		48(81.4)	11(18.6)		23(39.0)	36(61.0)	
pT3	12	8(66.7)	4(33.3)		6(50.0)	6(50.0)		1(8.3)	11(91.7)	
Lymph node metastasis				**0.004**			0.278			0.558
Positive	22	18(81.8)	4(18.2)		15(68.2)	7(31.8)		9(40.9)	13(59.1)	
Negative	59	27(45.8)	32(54.2)		47(79.7)	12(20.3)		20(33.9)	39(66.1)	
pTNM stage				**0.031**			0.093			0.757
I	43	23(53.5)	20(46.5)		37(86.0)	6(14.0)		17(39.5)	26(60.5)	
II	19	7(36.8)	12(63.2)		13(68.4)	6(31.6)		6(31.6)	13(68.4)	
III/IV	19	15(78.9)	4(21.1)		12(63.2)	7(36.8)		6(31.6)	13(68.4)	

NOTE: [0 ≤ score ≤ 1] and [2 ≤ score ≤ 4], which represented negative and positive of 14-3-3ζ, TGFβR1 and pSMAD3, respectively.

**Figure 2:**
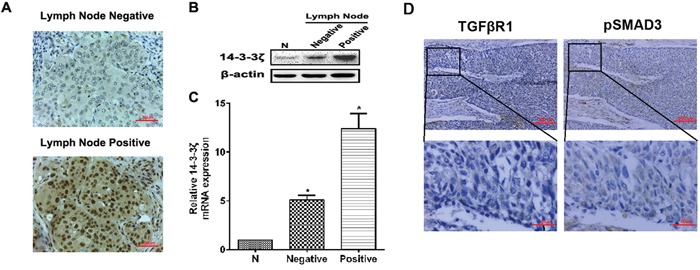
The expression of 14-3-3ζ, TGFβR1 and pSMAD3 proteins in lung SCC tissues and the relationship with clinicopathological characteristics **A.** The expression of 14-3-3ζ was evaluated in positive and negative lymph node metastasis by IHC. Original magnification: 40×. Representative images are shown. **B.** and **C.** Protein or RNA was extracted from lymph node metastasis of lung SCC patients or adjacent normal tissues and subjected to western blot or real-time PCR analysis to examine the level of 14-3-3ζ expression. β-Actin served as a loading control. All data are expressed as the means ± standard deviation (SD) for three independent experiments. N (adjacent normal tissue) served as a control. **D.** The expressions of TGFβR1 and pSMAD3 proteins were shown in the cytoplasm of cancer cells.

We also investigated 14-3-3ζ expression in lung SCC patients with different lymph node metastasis. 14-3-3ζ expression was higher expressed in lung SCC patients who had positive lymph node metastasis by IHC (Figure 2A). It also confirmed that both protein and RNA level, 14-3-3ζ was highly expressed in positive lymph node patients than in the negative lymph node patients (Figures 2B and 2C).

We assessed expression of TGFβR1 and pSMAD3. The rate of high expression of TGFβR1 and pSMAD3 were 76.5% and 35.8% respectively in lung SCC samples (n=81) (Table 1), which were primarily localized to the cytoplasm (Figure 2D). TGFβR1 expression was correlated with tumor size (*p*=0.002) and differentiation (*p*=0.006), but not with other clinical parameters. Similarly, pSMAD3 expression was closely associated with tumor size (*p*=0.023) (Table 1).

In addition, the results proved that the increased expression of 14-3-3ζ was associated with high levels of TGFβR1 (r=0.279, *p*=0.005), and pSMAD3 (r=0.216, *p*=0.033). Expression of TGFβR1 protein was also associated with pSMAD3 expression (r=0.313, *p*=0.002) in lung SCC tissues (Table 2).

**Table 2:** Association between 14-3-3ζ, TGFβR1, and pSMAD3 in 81 lung SCC tissues

		TGFβR1	pSMAD3
14-3-3ζ	Kendall's tau-b	0.279**	0.216*
	Sig. (2-tailed)	.005	.033
TGFβR1	Kendall's tau-b	1	0.313**
	Sig. (2-tailed)	NS	.002

* Correlation is significant at the 0.05 level (2-tailed).

** Correlation is significant at the 0.01 level (2-tailed).

### Association of 14-3-3ζ, TGFβR1 and pSMAD3 proteins with survival of lung SCC patients

**Figure 3:**
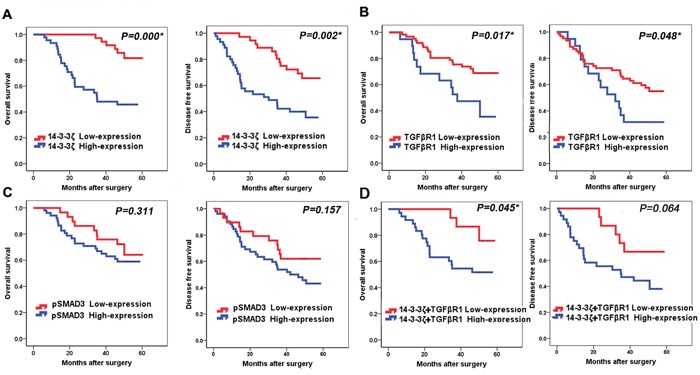
Association of 14-3-3ζ, TGFβR1 and pSMAD3 proteins with survival of lung SCC patients **A.** Kaplan-Meier curve analysis of association between OS, DFS and 14-3-3ζ expression in lung SCC patients. **B.** Kaplan-Meier curve analysis of association between OS, DFS and TGFβR1 expression in lung SCC patients. **C.** Kaplan-Meier curve analysis of association between OS, DFS and pSMAD3 expression in lung SCC patients. **D.** Kaplan-Meier curve analysis of association between OS, DFS and patients who had high expression of both 14-3-3ζ and TGFβR1with patients who had low expression of both 14-3-3ζ and TGFβR1 expression in lung SCC patients.

Kaplan-Meier curve analysis showed that the highexpression of 14-3-3ζ had a shorter overall survival (OS) than those with low 14-3-3ζ expression, and had a worse disease-free survival (DFS) (Figure 3A, *p*=0.000 and *p*=0.002, respectively). Furthermore, patients with high TGFβR1 expression had a shorter OS and DFS than those with low TGFβR1 expression (Figure 3B, *p*=0.017 and 0.048, respectively). However, high expression of pSMAD3 protein was not corelated to OS or DFS (Figure 3C, *p*=0.311 and 0.157, respectively). Furthermore, univariate analysis showed that 14-3-3ζ expression was significantly associated with OS (*p*=0.001; hazard ratio, 4.827; 95% CI, 1.966-11.854; Table 3). Multivariable analysis also confirmed that 14-3-3ζ expression was an independent prognostic factor for lung SCC patients (*p*=0.000, Table 3). Similarly, tumor TNM stage was also an independent prognostic factor for lung SCC patients (*p*=0.041, Table 3). Moreover, expression of TGFβR1 proteins was also predictors for OS in this cohort of patients.

**Table 3:** Prognostic factors for lung SCC patients

Variables	HR	Univariate 95% CI	*P*	HR^§^	Multivariate 95% CI	*P*^§^
OS						
Age (years)						
≤55 vs. >55	1.009	0.962-1.058	0.712			
Gender						
Male vs. female	0.563	0.171-1.855	0.345			
Differentiation						
Well/moderate vs poor	1.442	0.716-2.903	0.306			
pT status						
pT2-4 vs pT1	1.806	1.089-2.996	**0.022**			0.824
Lymph node metastasis						
Positive vs. Negative	1.884	1.238-2.865	**0.003**			0.463
pTNM stage						
II-III vs. I	2.070	1.446-2.962	**0.000**	1.878	1.026-3.438	**0.041**
14-3-3ζ expression						
low vs. high	4.827	1.966-11.854	**0.001**	8.358	3.120-22.391	**0.000**
TGFβR1 expression						
low vs. high	0.415	0.197-0.874	**0.021**	0.219	0.092-0.521	**0.001**
pSMAD3 expression						
low vs. high	0.671	0.307-1.466	0.317			

CI, confidence interval; HR, hazard ratio; OS, overall survival.

Lung SCC patients with high expression of both 14-3-3ζ and TGFβR1 have significantly shorter OS compared to patients with low 14-3-3ζ and TGFβR1 expression (*p*=0.045, Figure 3D).

### 14-3-3ζ promotes the proliferation of in lung SCC cells *in vitro*

**Figure 4:**
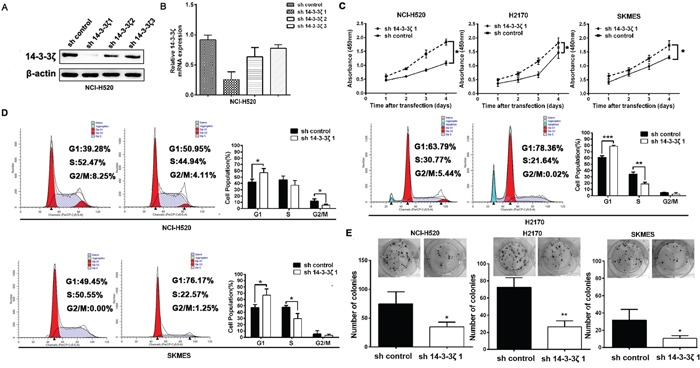
14-3-3ζ promotes the proliferation of in lung SCC cells *in vitro* **A.** and **B.** 14-3-3ζ expression was confirmed by western blot and real-time PCR. It was dramatically decreased by RNA interference in NCI-H520 cells by sh14-3-3ζ 1. **C.** CCK-8 assays were used to investigate the changes in proliferation rate of NCI-H520, H2170 and SKMES cells at different time intervals ranging from 24 to 96 h. Mean ± SD of absorbance from each group (n =3) is shown. **D.** Cell cycle analysis was determined by flow cytometry. **E.** Colony-forming efficiency was detected in NCI-H520, H2170 and SKMES cells treated with 14-3-3ζ-shRNA.

To gain the potential role of 14-3-3ζ as a poor prognostic biomarker being associated with lung tumor proliferation, lung SCC cells, H520, H2170 and SKMES cells were transfected with shRNAs against 14-3-3ζ, which were referred to as sh14-3-3ζ 1, sh14-3-3ζ 2, and sh14-3-3ζ 3. sh14-3-3ζ 1 was the most effective among the three shRNA constructs verified by real-time PCR and western blot (Figure 4A, 4B). Therefore, the stable cell lines transfected with sh14-3-3ζ 1 were used in the following experiments.

Compared with control, sh-14-3-3ζ significantly inhibited cell proliferation in H520, H2170 and SKMES cells *in vitro* proliferation assay (Figure 4C). Accordingly, we decided to analyze the roles of 14-3-3ζ in cell cycle phases contributes to tumor proliferation. As seen in Figure 4D, sh14-3-3ζ cells were decreased G1/S transition. In addition, colony formation analysis showed that the colony-forming efficiency of sh14-3-3ζ were significantly lower than that of cells with sh-control (*p*<0.05, Figure 4E).

### 14-3-3ζ induces the migration, invasion and regulates EMT marker of in lung SCC cells *in vitro*

**Figure 5:**
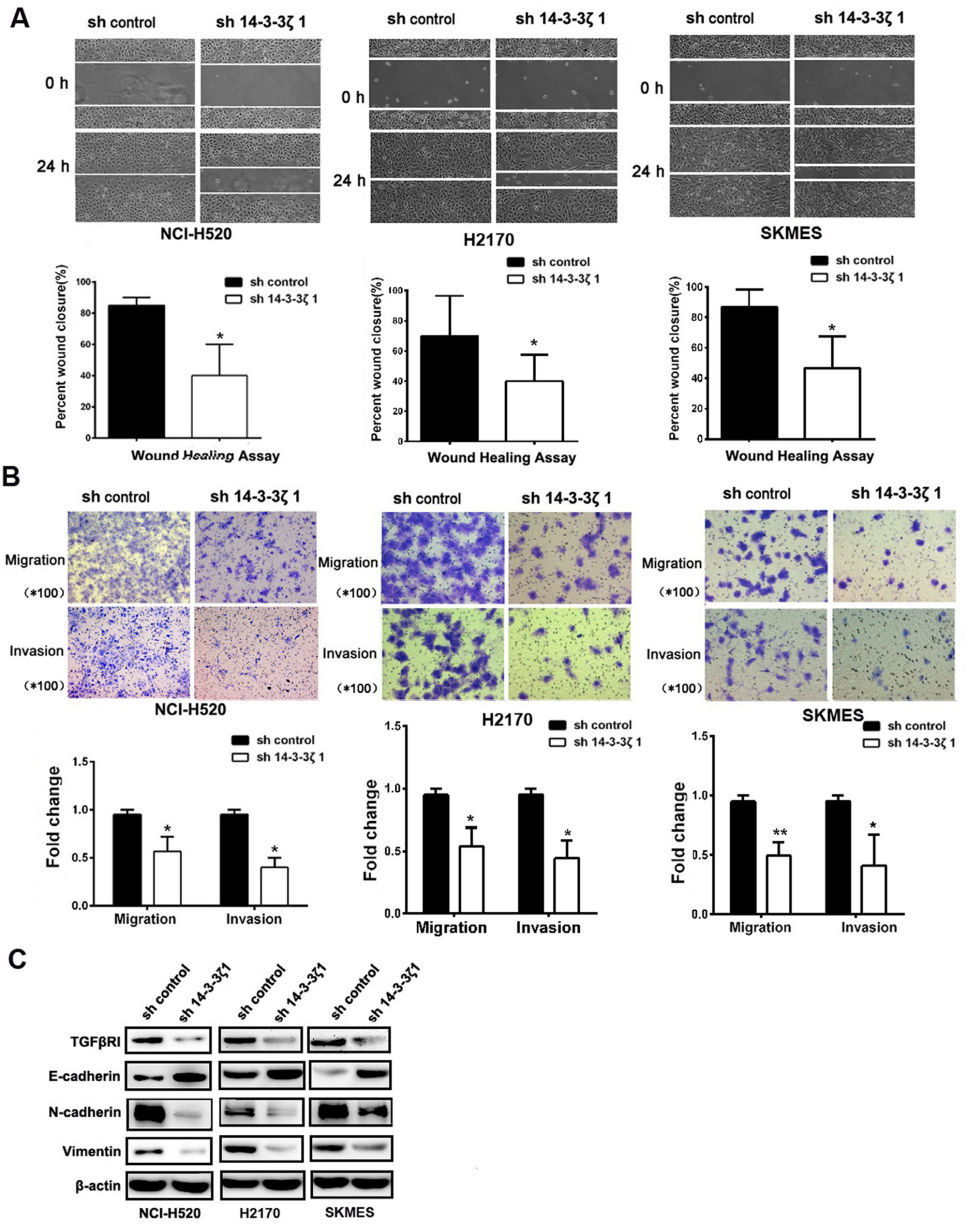
14-3-3ζ induces the migration, invasion and regulates EMT marker of in lung SCC cells *in vitro* **A.** Wound-healing assays were used to investigate themigratory ability of NCI-H520, H2170 and SKMES cells. **B.** The invasion and migration ability of three cell lines and their derivatives was determined by Transwell assay. All data are expressed as the means ± standard deviation (SD) for three independent experiments. **C.** Analysis of TGFβR1, E-cadherin, N-cadherin, and Vimentin in NCI-H520, H2170 and SKMES cells treated with 14-3-3ζ-shRNA by western blot.

To investigate the potential role of 14-3-3ζ in tumor metastasis, using a wound healing assay, it showed that cells transfected with sh14-3-3ζ resulted in a slower closing of scratch wounds compared with that of control group (Figure 5A). Transwell assay showed that 14-3-3ζ knockdown suppresses migration and invasion ability in lung SCC cells compared to the control (Figure 5B).

EMT has been considered as an important mechanism that accelerates cancer cell migration and leads to metastasis, so we also investigated whether 14-3-3ζ is involved in the EMT to promotion cancer metastasis. E-cadherin, N-cadherin and Vimentin, putative EMT-related markers, were evaluated by Western Blotting. Knockdown of 14-3-3ζ inhibited the expression of Vimentin and N-cadherin, but partially rescued the expression of E-cadherin (Figure 5C). These observations indicate that 14-3-3ζ can also suppress the EMT of lung SCC. Collectively, our findings suggest that 14-3-3ζ negatively regulate tumor migration, invasion and EMT marker expression of lung SCC cells. Furthermore, the knockdown of 14-3-3ζ inhibited the expression of TGFβR1 (Figure 5C).

### 14-3-3ζ regulates proliferation, metastasis and EMT markers through TGFβR1

**Figure 6:**
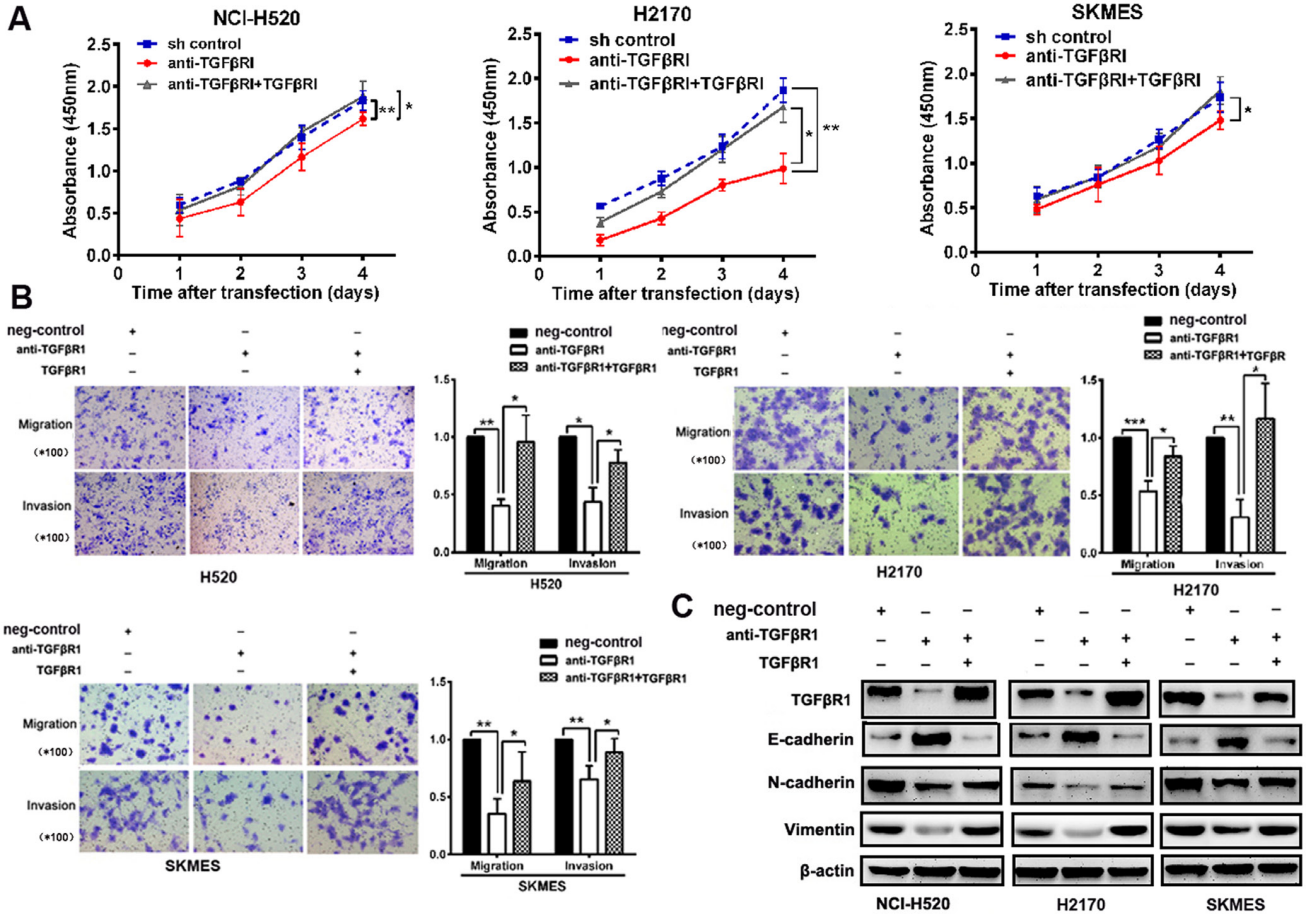
14-3-3ζ regulates proliferation, metastasis and EMT markers through TGFβR1 **A-C.** Through the knock-down TGFβR1 in lung SCC cells, cells were decreased proliferation, migratory and invasive capability and inhibited EMT markers than control. Through transfection of TGFβR1 in cells, cells were increased proliferation, migratory, invasive capability and EMT markers expression than control.

Through knockdown TGFβR1 in lung SCC cells, cells were decreased proliferation, migratory and invasive capability and inhibited EMT markers than control. Through transfection of TGFβR1 in TGFβR1-knockdown cells, cells were increased proliferation, migratory, invasive capability and EMT markers expression than control (Figures 6A-6E). These results indicated that TGFβR1 has a critical role in 14-3-3ζ -induced cell proliferation and metastasis of lung SCC cells.

### Knockdown of 14-3-3ζ suppressed tumor growth and decreases the formation of metastases *in vivo*

**Figure 7:**
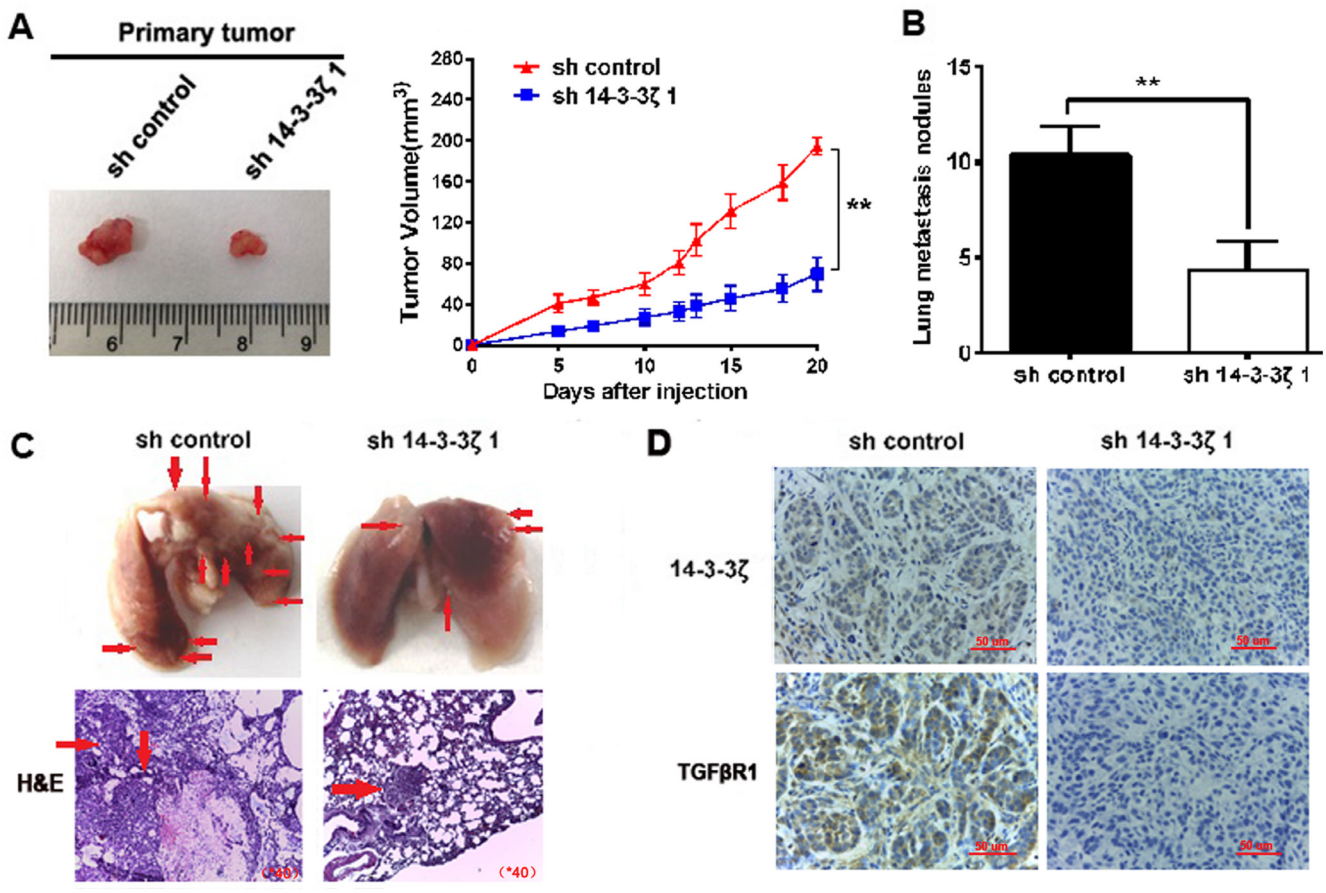
Knockdown of 14-3-3ζ suppressed tumor growth and decreases the formation of metastases *in vivo* **A.** Knockdown of 14-3-3ζ remarkably reduced the volumes of tumor mass. Volumes of tumors formed by NCI-H520/shcontrol or NCI-H520/sh14-3-3ζ1 stable clones in nude mice were measured. Left shows the gross view of isolated tumors. Right shows the mean ±SD (n = 6) from each group at each time point. **B.** The number of metastatic nodules in the lung was decreased in the sh14-3-3ζ group compared to the sh-control group. **C.** Images of representative lung metastasis in the different groups. **D.** sh-14-3-3ζ could significantly decrease the expression of TGFβR1 *in vivo*.

To further verify if 14-3-3ζ silencing inhibits tumor proliferation *in vivo*, NCI-H520/sh-control or NCI-H520/sh14-3-3ζ#1 stable clones were established and injected into the ventral region of nude mice. As shown in Figure 7A, we found that knocking down 14-3-3ζ in NCI-H520 dramatically suppressed its growth *in vivo*.

We also investigated if 14-3-3ζ decreases lung metastasis *in vivo*. The numbers of lung metastasis nodules were significantly reduced in sh14-3-3ζ group compared to control group (Figure 7B). The representative images of lung metastasis are shown in Figure 7C.

To clarify the mechanisms underlying 14-3-3ζ-mediated tumor growth and metastasis, we further verified the expression of TGFβR1 using resected tissues from xenograft tumors. Knockdown of 14-3-3ζ could significantly decrease the expression of TGFβR1 *in vivo* (Figure 7D). These results indicated that 14-3-3ζ promoted the proliferation and metastasis of lung SCC.

## DISCUSSION

Metastasis is the main leading causes of mortality of lung SCC for lacking of effective prediction marker for monitoring of disease progression or prognosis. Therefore, it is urgent to elucidate the molecular mechanisms of metastasis and prognosis underlying lung SCC. In previously study, 14-3-3ζ was over expression in lung cancer tissues and was positively associated with stage and grading of NSCLC [14, 15]. 14-3-3ζ can be an independent prognostic factor for predicting outcome in early-stage patients in NSCLC. In this study, we demonstrated that the expression of 14-3-3ζ was high expressed in human lung SCC tissues compared with its normal tissues and increased with progress of TNM stage. These results shown that 14-3-3ζ may be involved in tumorgenesis of lung SCC.

Recently, 14-3-3ζ was reported to have effects on tumor EMT and metastasis [5, 9, 16, 17]. In this study, high expression of 14-3-3ζ was correlated to pTNM stage and lymph node metastasis in lung SCC. Additionally, lung SCC patients with high 14-3-3ζ expression had a worse prognosis than those with low expression. Knockdown of 14-3-3ζ expression significantly inhibited the migratory and invasive capacities of NSCLC cells *in vitro*. Sh-14-3-3ζ also significantly reduced the lung metastases *in vivo*. These results indicated that 14-3-3ζ was a negative regulator in lung SCC invasion and metastasis.

Tumor metastasis is a complex process, starting with primary tumor invasion through endothelial barriers known as epithelial-to-mesenchymal transition (EMT), characterized by loss of cell-cell adhesion and increased cell motility [18–19]. TGFβR1 is a ubiquitin-dependent degradation protein and a pivotal protein in the EMT signaling pathway [20]. In breast MCF-10A cell lines, 14-3-3ζ could bound to TGFβR1 and inhibit the proteasome-mediated TGFβR1 degradation [13]. In this study, 14-3-3ζ expression was positively correlated with levels of TGFβR1 and pSMAD3 expression. The expression of TGFβR1 was positively correlated with a high level of pSMAD3 protein. Lung SCC patients with high expression of both 14-3-3ζ and TGFβR1 have significantly shorter OS compared to patients with low 14-3-3ζ and TGFβR1 expression. We also found that TGFβR1 was responsible for cell metastasis of SCC cells after regulating 14-3-3ζ expression. These results indicated that TGFβR1 has a critical role in 14-3-3ζ -mediated cell proliferation and metastasis of lung SCC cells.

Prior study showed that 14-3-3ζ impaired cell proliferation in human ectopic endometrial *in vitro* [21]. 14-3-3ζ dysregulates BH3-only protein and leads to a lower level of Bax activation resulting in apoptosis suppression [22–23]. Furthermore, 14-3-3ζ can interact with a tumor suppressor Tuberin via Akt phosphorylation [24], thereby inducing hyperactivation of the PI3K/Akt pathway and downregulation of p53 [25]. In addition, 14-3-3ζ can suppress the function of Raf-1 in tumorigenesis through Raf CRD [26]. Our work demonstrated that high levels of 14-3-3ζ can promote lung SCC cell proliferation *in vitro* and *in vivo*. Furthermore, we showed that 14-3-3ζ accelerated G1/S transition to promote lung cancer cell cycle progression and cell proliferation. TGFβR1 was responsible for 14-3-3ζ-mediated cell proliferation and metastasis in lung SCC. Thus we have identified a novel function of 14-3-3ζ protein, which is responsible for growth-promotion and cell cycle regulation.

In the light of the above findings, 14-3-3ζ can be considered as a novel marker of metastasis and prognosis in lung SCC patients. Our study not only reveals the pathological role of 14-3-3ζ in lung SCC, but also illuminates promising prognostic biomarkers and therapeutic target for lung SCC.

## MATERIALS AND METHODS

### Cell lines and culturing

The lung SCC cells, NCI-H520, H2170 and SKMES cells were obtained from Heilongjiang Cancer Institute (Harbin, China). Cells were cultured in 1640 medium with 10% fetal bovine serum. Cells were maintained at 37°C with 5% CO2.

### Immunohistochemistry

We obtained 81 cases tissue specimens from lung SCC patients who underwent surgery between December 2008 and June 2010 in Harbin Medical University Cancer Hospital. 27 pairs of lung SCC tumors and its adjacent normal tissues were chosed from 81 lung SCC patients. Tissue sections were immersed in MEDTA, bathed in a steamer at 100°C for 15 min and maintained in methanol for 15 min. The slides were incubated with for 14-3-3ζ primary antibody (Santa Cruz Biotechnology, #sc-1019, diluted at 1:30), TGFβR1 (Santa Cruz Biotechnology, #sc-398, diluted at 1:75) and pSmad3 (Santa Cruz Biotechnology, #sc-130218, diluted at 1:75), stained using DAB and counterstained using hematoxylin. The relative staining intensity was scored as 0 (0%), 1 (1 to 25%), 2 (26 to 50%), 3 (51 to 75%), and 4 (76 to 100%), according to the percentages of immunoreactive cells. The score ≤1 was defined as low expression, and the score ≥2 was defined as high expression.

### Western blot analysis

The cells or tissues were lysed in lysis buffer. 50 μ g of protein sample each was loaded on SDS-PAGE (10% gels) and transferred onto polyvinylidene fluoride (PVDF) membrane. The proteins were probed with 14-3-3ζ, TGFβR1, E-cadherin (Santa Cruz Biotechnology, #sc-7870), N-cadherin (Abcam, ab18203), Vimentin antibody (Abcam, ab92547). The bound antibodies were detected using ECL Western Blotting Detection system. β-actin was used as the loading control (Sigma, A1978).

### Real time-PCR

RNA from cells and tissues were isolated using Trizol method (Invitrogen). RNAs were then reverse-transcribed into cDNA using cDNA Synthesis Kit (Fermentas, Foster City, CA). Levels of RNA expression were determined by SYBR Premix Ex-Taq II Kit and an ABI 7500 machine according to manufacturer's instructions. 2^−ΔΔCt^ method was used to calculated the results. The β-actin was as an internal control.

### Plasmid transfection

shRNA-14-3-3ζ plasmids (#1, 2, and 3 nucleotides) or the controls were purchased from Santa Cruz Biotechnology. The three sequences for 14-3-3ζ were selected:

5′-GCCUGCAUGAAGUCUGUAATTUUACAGACUUCAUGCAGGCTT-3′,

5′-CGUCUCAAGUAUUGAACAATTUUGUUCAAUACUUGAGACGTT-3′ and

5′-CACGCUAAUAAUGCAAUUATTUAAUUGCAUUAUUAGCGUGTT-3′. PLKO vector (Invitrogen, St. Louis, MO, USA) was used. Infected cells were selected by puromycin (1.25 ug/ml).

### Wound-healing assay

1×10^6^/ml cells were seeded in six-well plates. 200 μl sterile pipette tip was used to scratch the wounds. The cells were stained and rinsed with PBS. Images of cells at the edge of the scratch was obtained at time 0 and 24 h.

### Cell migration and invasion assay

Cells suspended in medium without serum were seeded into the upper chamber of transwell (Corning). Cells were incubated for 24h. The surface of the chamber membrane were cleaned, fixed with 90% ethanol, stained with crystal violet and photographed using microscope at×400 magnification.

### Cell proliferation assay

Briefly, 5×10^3^ cells per well were seeded in 96-well plates, and incubated for 24, 48, 72, or 96h. The absorbance at 450nm was measured after incubation with 10 μl of Cell Counting Kit-8 (CCK-8) assay reagent (Dojindo, Kumamato, Japan) for 1h at 37°C by BioTek ELx800 microplate photometer (SN211805, US). The assay was performed three times.

### Flow cytometry

Cells were washed with cold PBS and fixed with 70% ethanol overnight at −20°C for cell cycle analysis. Fixed cells were washed with PBS for 10 min and treated with PI/RNase staining. Cell cycle for each sample was evaluated by flow cytometer (Becton-Dickinson) following the manufacturer's instructions. The profile of cell cycle was analyzed with MultiCycle software (Phoenix Flow Systems).

### Colony formation assay

The transfected cells were diluted and replaced in six-well plates. Incubating for 10 days, cells were treated with PBS and fixed using methanol. Then cells were stained with crystal violet, counted and calculated. The experiments were conducted at least three times.

### Xenograft models

The animal experiments were approved by the Scientific Investigation Board of tumor Hospital of Harbin Medical University. 4-6 weeks old nude mice (male) were purchased from the Shanghai Experimental Animal Center and bred in Animal Center of the affiliated tumor hospital of Harbin Medical University. Six mice were injected with 1×10^6^ H520/sh control or H520/sh14-3-3ζ cells respectively. Their tumor volume (V) was calculated with the equation: V= (W^2^×L) ×0.5.

For tumor metastasis analysis, nude mice were inoculated with 1× 10^6^ H520/sh control or H520/sh14-3-3ζ cells via tail vein injection. Four or seven weeks later, mice were sacrificed, and tumor and their lungs were harvested and fixed in formalin.

### Statistical analysis

SPSS 18.0 and Graphpad Prism software were used for statistical analyses. The statistical significance between two groups was calculated by Student's t-test. The association between 14-3-3ζ, TGFβR1 and pSmad3 expression and tumor characteristics were evaluated by x^2^ test and Cox regression. Data were expressed as mean ±SD. The statistical significance is described using asterisks (*). * = *p* <0.05; ** = *p* <0.01; *** = *p* <0.001.
